# High expression of ezrin predicts poor prognosis in uterine cervical cancer

**DOI:** 10.1186/1471-2407-13-520

**Published:** 2013-11-04

**Authors:** Jienan Kong, Yan Li, Shuangping Liu, Haidan Jin, Yongjun Shang, Chengshi Quan, Yulin Li, Zhenhua Lin

**Affiliations:** 1 Key Laboratory of Natural Resources of Changbai Mountain & Functional Molecules (Yanbian University), Ministry of Education, Yanji, China; 2 Department of Pathology, Yanbian University Medical College, Yanji 133002, China; 3 Department of Pathology, Luhe Teaching Hospital of the Capital Medical University, Beijing 101149, China; 4 Department of Orthopedics, Affiliated Hospital of Chifeng University, Chifeng 024000, China; 5 The Key Laboratory of Pathobiology, Ministry of Education, Bethune Medical College, Jilin University, Changchun 130021, China

**Keywords:** Uterine cervical cancer, Ezrin, Human papillomavirus, Survival analysis

## Abstract

**Background:**

Ezrin, a member of the ezrin/radixin/moesin (ERM) protein family, plays a pivotal role in tumor invasion and metastasis. This study is aimed to investigate the clinicopathological significance of upregulated ezrin protein expression in uterine cervical cancers.

**Methods:**

Immunohistochemical staining of ezrin protein was performed on uterine cervical cancer specimens from 235 patients. For comparison, 239 cases of cervical intraepithelial neoplasia (CIN), 17 cases of cervical glandular intraepithelial neoplasia (CGIN) and 52 normal cervix samples were also included. qRT-PCR was performed on fresh tissues to detect ezrin mRNA expression levels. HPV infection statuses were genotyped by oligonucleotide microarray, and 10-year survival rates were calculated using the Kaplan-Meier method for 109 cervical cancer patients.

**Results:**

Apical membranous distribution of ezrin protein was only observed in normal cervical glands, while perinuclear staining was only observed in cervical cancers. Strong cytoplasmic and diffuse localization of ezrin were frequently seen in the cervical cancers compared with the normal counterparts. Furthermore, this strongly positive ezrin expression was significantly higher in cervical cancers than in CIN, CGIN, and normal cervical epithelia. Ezrin overexpression was closely related with poor differentiation, late stage, and lymph node metastasis. Additionally, ezrin overexpression was associated with lower 10-year survival rate for patients with early stage cervical cancer, but not for patients with advanced stage.

**Conclusions:**

Aberrant localization and overexpression of ezrin might be an independent effective biomarker for prognostic evaluation of cervical cancers.

## Background

It is well known that tumor metastasis is a multi-gene and multi-step process, and each of these steps involves substantial interactions between neoplastic cells and adjacent non-neoplastic tissues [1]. Currently, increased evidence suggests that a membrane-cytoskeleton linker, ezrin, plays a pivotal role in tumor invasion and metastasis [2]. Ezrin, which is encoded by the *EZR* gene located at chromosome 6q25.2–q26, is a member of the ezrin/radixin/moesin (ERM) protein family and is a membrane cytoskeletal binding protein. *EZR* is the most widely studied gene of this family, and was initially thought to be a simple cross-linker between actin filaments and other membrane proteins [3-5]. Furthermore, ezrin is known to function as an organizer of cell-cell adherent junctions and may play an important role in the metastasis of epithelial neoplasms. It can maintain cell polarity, is involved in cell movement and adhesion between cells and the extracellular matrix (ECM), regulates cell immune function, and is related to cell senescence and death. Most importantly, these characteristics are all closely related to cancer development. Ezrin may have important functions in plasma membrane structures other than microvilli, and it is known that active ezrin is associated with these structures through its N-terminus [6]. Ezrin was also demonstrated to co-precipitate with β-catenin and E-cadherin, key proteins involved in cell adhesion [7]. Overexpression of ezrin protein in variety of tumors, such as carcinomas of the endometrium [8,9], ovary [10] and pancreas [11,12], has been shown to enhance metastatic potential. Furthermore, Khanna et al. [13] found high ezrin expression in osteosarcomas was associated with early development of metastasis. Consistent with these reports, suppression of ezrin protein expression and disruption of its function significantly reduced lung metastasis in a mouse osteosarcoma model [14]. Li et al. found that ezrin silencing by small hairpin RNA could reverse the metastatic behavior of human breast cancer cells, indicating an important role for ezrin in regulating tumor metastasis and progression [15].

Nevertheless, studies to date have not systematically explored the relationship between ezrin and its clinicopathological significance in cervical cancers, particularly the correlation between ezrin expression and human papillomavirus (HPV) infection. Thus, we aimed to analyze the expression and localization of ezrin in cervical cancers compared with precancerous disease and normal cervical epithelia, determine its relationship with clinicopathological parameters, and investigate its prognostic value for cervical cancer patients based on tumor stage and survival data. Additionally, HPV infection was examined to investigate its correlation with ezrin expression in cervical cancers.

## Methods

### Ethics statement

This study complied with the Helsinki Declaration and was approved by the Human Ethics and Research Ethics committees of Yanbian University Medical College in China. Through the surgery consent form, patients were informed that the resected specimens were stored by our hospital and potentially used for scientific research, and that their privacy would be maintained. Follow-up survival data were collected retrospectively through medical-record analyses.

### Tissue specimens

Routinely processed and diagnosed uterine cervical lesion tissues were selected from the Department of Pathology and Tumor Tissue Bank, Yanbian University Medical College, and included 52 non-neoplastic cervical epithelia samples, 239 cervical intraepithelial neoplasms (CIN; CIN-1, n=65; CIN-2, n=102; CIN-3, n=72), 17 cervical glandular intraepithelial neoplasms (CGIN), 226 squamous cervical cancers (SCCs), and nine adenocarcinomas (AC). All cervical tissue specimens were selected from punch biopsies, loop electrosurgical excisions, cone biopsies and hysterectomies, and all 52 non-neoplastic cervical tissues were obtained from leiomyoma patients who underwent hysterectomies. Specimens were obtained between 1995 and 2009, and the cancer patients were aged 23–79 years. All SCC and AC tumor specimens were obtained from pretreatment surgical resections, and the data were retrieved from patients’ operative and pathological reports. Staging was performed according to the TNM and FIGO classification of carcinomas of the uterine cervix; 108 were considered early stage (FIGO stages I–IIA) and 127 advanced stage (IIB–IV), according to the Union for International Cancer Control (UICC) criteria 7th Edition and WHO classification [16]. A total 109 of cervical cancer patients had follow-up records for more than 10 years, and the follow-up deadline was November 2011. The survival time was counted from the date of surgery to the follow-up deadline, or date of death (usually the result of cancer recurrence or metastasis). H&E stained slides were reviewed by two experienced pathologists.

### Immunohistochemical staining for ezrin in paraffin-embedded tissues

For immunohistochemical studies with a DAKO LSAB kit (DAKO A/S, Glostrup, Denmark), 4 μm-thick tissue sections were deparaffinized, rehydrated and incubated with 3% H_2_O_2_ in methanol for 15 minutes at room temperature to eliminate endogenous peroxidase activity. Antigen retrieval was performed by placing the slides in 0.01M sodium citrate buffer (pH 6.0) at 95°C for 20 minutes. After overnight incubation at 4°C with primary antibody against ezrin (1:50, #3145; Cell Signaling Technology, Boston, USA), sections were treated according to standard immunoperoxidase methods using a streptavidin-biotin peroxidase complex kit (LSAB+Kit/HRP, DAKO). The peroxidase reaction was developed with 3,3’-diaminobenzidine (DAB), then counterstained with Mayer's hematoxylin. Rabbit IgG isotope used as a negative control and positive tissue sections were processed omitting the primary antibody as a further negative control.

### Evaluation of immunohistochemical staining

All slides were evaluated independently by two pathologists without prior knowledge of clinical outcome. We first observed the staining in the whole cervical epithelium, and then quantified 50 representative fields. The interpretation criteria were described previously by Elzagheid A et al. [17] and Jin J et al. [18]. Briefly, the immunostaining of ezrin was semi-quantitatively scored as ‘-’ (negative, no or less than 5% positive cells), ‘+’ (5–25% positive cells), ‘++’ (26–50% positive cells) and ‘+++’ (more than 50% positive cells). The strongly positive descriptor (ezrin overexpression) was assigned to ‘++’ and ‘+++’ scored cells. For survival analysis, ezrin expression level was denoted as high expression (‘++’ and ‘+++’) and low expression (‘-’ and ‘+’).

### HPV genotyping by oligonucleotide microarray (HPV-DNA chip)

DNA was extracted from the paraffin-embedded cervical lesion specimens using the High Pure PCR Template Preparation Kit (Cat.11796828001, Roche, Penzberg, Germany), and HPV detection and genotyping were performed using a PCR-based HPV-DNA microarray system (Biomedlab, Seoul, South Korea). HPV-DNA chips can detect 22 types of HPV, including 15 high-risk types (HPV16, 18, 31, 33, 35, 39, 45, 51, 52, 56, 58, 59, 66, 68, 69) and seven low-risk types (6, 11, 34, 40, 42, 43, 44). Target HPV-DNA was amplified by PCR using primers (forward, 5′-tttkttachgtkgtdgatacyac-3′; reverse, 5′-gaaahataaaytgyaadtcataytc-3′; k, g/t; h, t/a/c; d, a/t/g; y, t/c) and labeled with Cy5-dUTP (NEN, life Science Products, MA, USA). β-globin at 110 bp was amplified with the primers detailed above as an internal control. The assay was performed according to the manufacturer’s protocol (GSI Lumonics, Scanarray Lite, Ottawa, Canada), as described previously [19].

### RNA extraction and quantitative real-time polymerase chain reaction (qRT-PCR)

Total RNA was extracted using Trizol reagent (Invitrogen, Carlsbad, CA) from seven fresh tissue samples of normal cervical epithelia, 15 of squamous cell carcinoma and 10 of adenocarcinoma. First-strand cDNA was synthesized by PrimeScript reverse transcriptase (TaKaRa Bio, Dalian, China) and oligo (dT) following the manufacturer’s instructions. To examine expression, real-time PCR was performed with a Bio-Rad sequence detection system according to the manufacturer’s instructions using double-stranded DNA-specific SYBR Premix Ex TaqTM II Kit (TaKaRa Bio). Double-stranded DNA specific expression was tested by the comparative Ct method using 2^-ΔΔCt^. Ezrin primers were as follows: 5′-TGGAGTTGATGCCCTTGGAC-3′; 5′-AGTCAGGTGCC TTCTTGTCG-3′. GAPDH: 5′-CATCACCATCTTCCAGGAGCG-3′; 5′-TGACC TTGCCCACAGCCTTG-3′. All assays were performed in triplicate at least three times.

### Statistical analysis

Chi-square tests were used to analyze the univariate associations of clinicopathological features with ezrin expression status. Survival curves were calculated using the Kaplan-Meier method in each group of patients with early stage and advanced stage cancer, and differences were analyzed using the log-rank test. The statistical significance of each test was set at *P*<0.05. All analyses were performed using the SPSS 17.0 statistical package (SPSS, Inc., Chicago, IL, USA).

## Results

### Atypical localization of ezrin protein in cervical lesions

**Figure 1:**
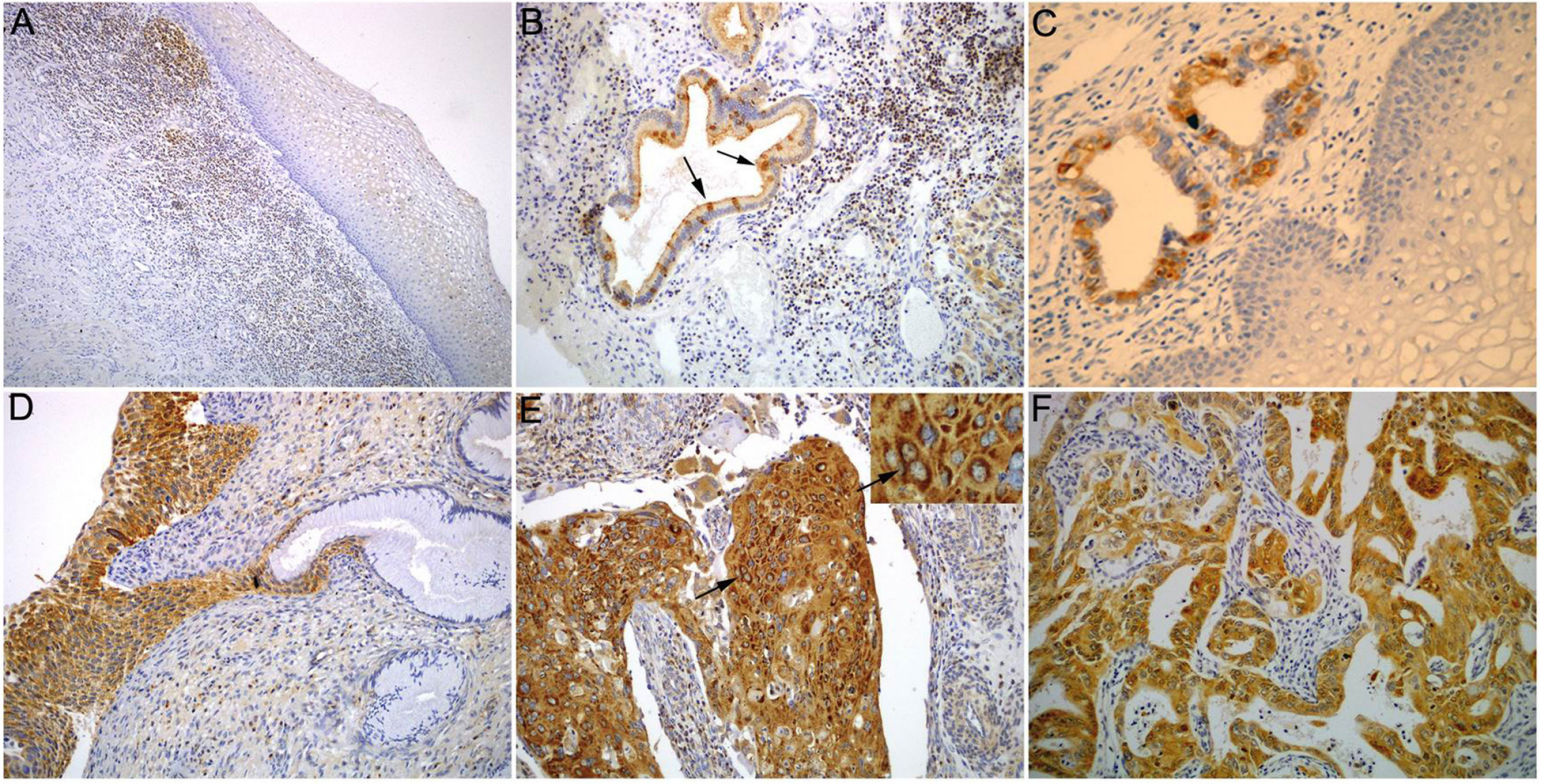
**Ezrin protein expression and localization in uterine cervical lesions. (A)** Ezrin protein was not expressed in normal cervical squamous epithelia, but showed strongly positive staining in the lymphocytes of the cervical stroma. **(B)** Scattered cells positive for ezrin were observed in normal glands; expression was concentrated at the apical membranes (*arrows*). **(C)** Ezrin protein was strongly expressed in the atypical glandular cells of CGIN, but was negative in the adjacent normal squamous epithelia. **(D)** Ezrin was expressed in the atypical cells of CIN-3, but not in the adjacent normal glandular cells. **(E)** Ezrin protein was strongly expressed in the cytoplasm of cervical squamous cell carcinomas and also exhibited perinuclear staining (*arrows*) (*insert*). **(F)** Ezrin protein was strongly expressed in the cytoplasm of cervical adenocarcinomas. (Original magnification, 200× in **A–F**, and 400× in *insert*).

**Figure 2:**
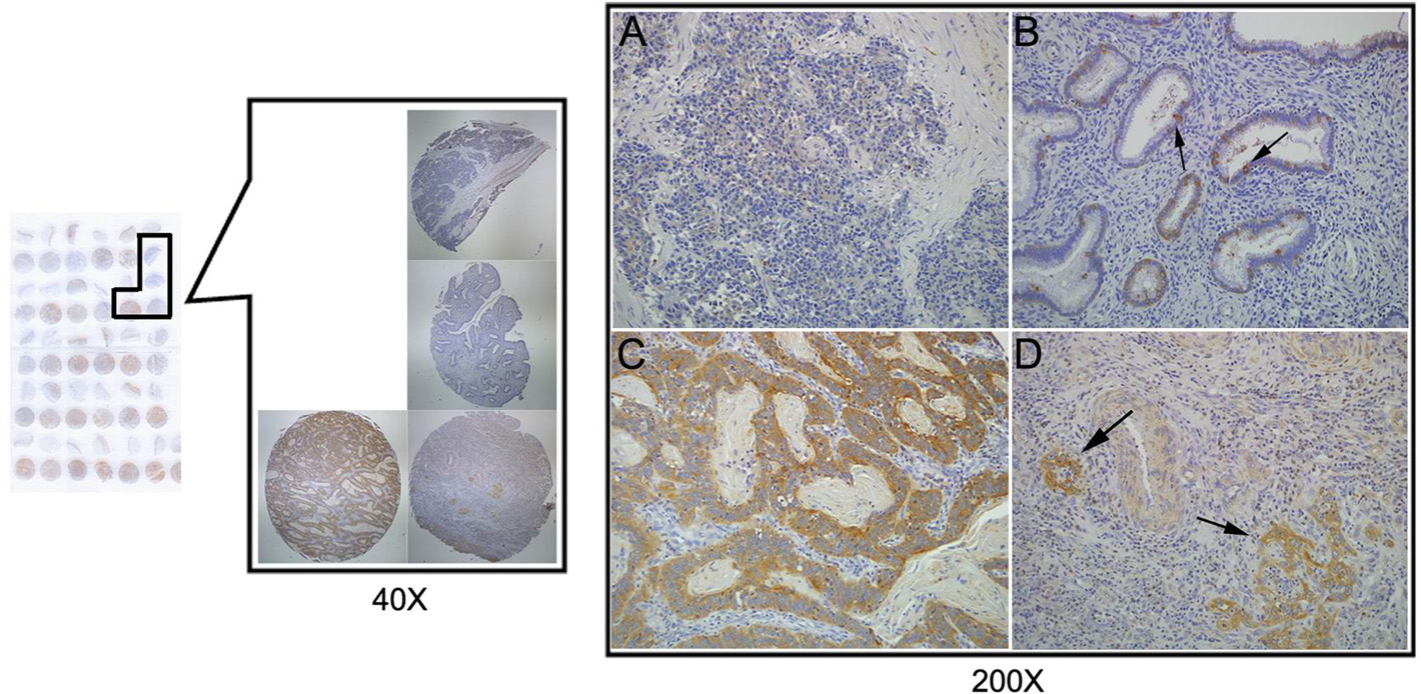
**Immunohistochemical staining for ezrin in tissue arrays of cervical lesions. (A)** Ezrin was not expressed in cervical squamous cell carcinoma. **(B)** Scattered apical membranous staining pattern (*arrows*) was observed in normal cervical glands. **(C)** Ezrin was moderately expressed in the cytoplasm of cervical adenocarcinoma cells. **(D)** Moderate ezrin expression in the invasive cancer loci (*arrows*) of cervical squamous cell carcinoma. (Original magnification, 200× in **A–D**).

Ezrin protein was negative in the squamous epithelial and glandular cells of most of the normal cervix cases (Figure 1A&B). Interestingly, some normal glands showed scattered ezrin staining at the apical membranes of normal glandular epithelial cells (Figure 1B). However, the dysplastic (CIN & CGIN) and cancer (SCC & AC) cells showed diffuse and strong cytoplasmic ezrin expression (Figure 1C–F), and the perinuclear staining pattern was only observed in SCC and AC, indicating that its distribution pattern might be helpful for early diagnosis of cervical cancers and their precancerous diseases. Additionally, ezrin cellular localization was compared with the clinicopathological features of cervical cancers: perinuclear staining of ezrin protein was only observed in cervical SCC and AC (51.4%, 108/210), but not in normal cervix and precancerous disease (Figures 1 and 2, Additional file 1: Table S1). In particular, ezrin perinuclear localization showed a positive association with higher differentiation (75.3% in well differentiated, 44.2% in moderately differentiated, and 21.2% in poorly differentiated cervical cancers) and early stage cervical cancers (79.3% in early stage and 31.7% in advanced stage of cervical cancers) (Additional file 2: Table S2); however, apical membranous distribution of ezrin protein was only observed in normal cervical glands, but not in cervical cancer and its precancerous disease.

### Quantification of ezrin overexpression in cervical cancers by immunohistochemistry and qRT-PCR

We first performed immunohistochemistry for ezrin protein in 543 samples of paraffin-embedded cervical lesions, and found that the positive rate of ezrin protein was only 11.5% (6/52) in non-neoplastic cervical tissues, but higher in CIN lesions (85.4%, 204/239), in which it was 81.5% (53/65) in CIN-1, 84.3% (86/102) in CIN-2 and 90.3% (65/72) in CIN-3. Also, the positive rate was higher in both SCC (88.9%, 201/226) and AC (100%, 9/9) of the cervix (*P*<0.01). However, the strongly positive rates of ezrin expression were absolutely negative in normal cervical epithelia (0%, 0/52), and lower in CIN (13.8% in CIN-1, 12.7% in CIN-2, and 22.2% in CIN-3) and CGIN (35.3%), but significantly higher in SCC (73.9%) and AC (88.9%) of the cervix (*P*<0.01) (Figure 1, Table 1).

**Table 1:** Ezrin protein expression in cervical lesions

**Diagnosis**	**Cases (n)**	**Ezrin**				**Positive rate (%)**	**Strongly positive rate (%)**
		**-**	**+**	**++**	**+++**		
Normal cervix	52	46	6	0	0	11.5%	0
CIN-1	65	12	44	9	0	81.5%**	13.8%
CIN-2	102	16	73	13	0	84.3%**	12.7%
CIN-3	72	7	49	16	0	90.3%**	22.2%
CGIN	17	0	11	6	0	100%**	35.3%
SCC	226	25	34	96	71	88.9%**	73.9%**
AC	9	0	1	4	4	100%**	88.9%**

**P<0.01 Compared with the normal cervix.

CIN: *Cervical Intraepithelial Neoplasia*; CGIN: *Cervical Glandular Intraepithelial Neoplasia.*

SCC: *Squamous cell carcinoma*; AC: *Adenocarcinoma.*

**Figure 3:**
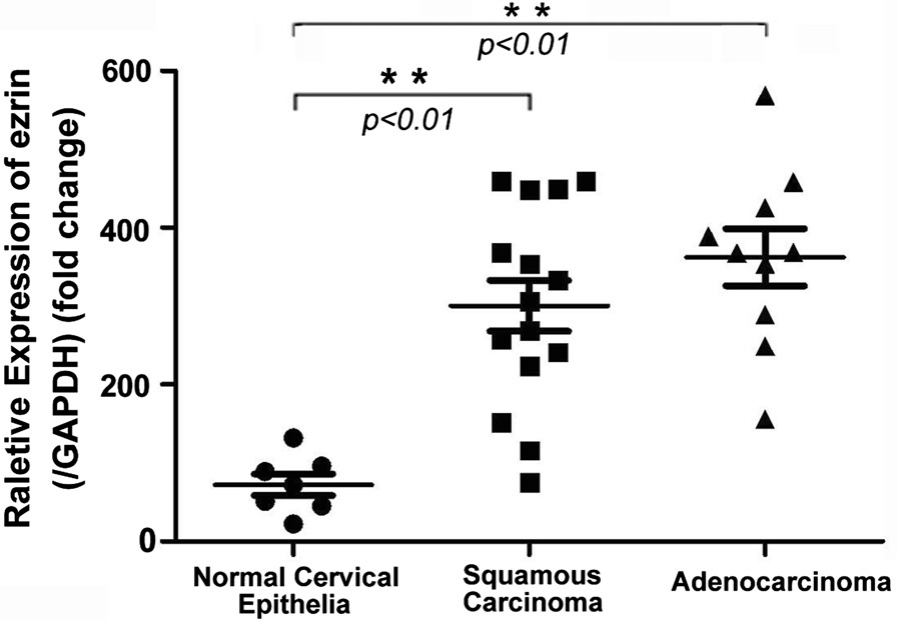
**qRT-PCR analysis of ezrin mRNA expression in normal cervical epithelia and cervical cancers.** Experiments were performed in triplicate for each case. Ezrin mRNA expression levels were significantly higher in cervical squamous cell carcinomas and adenocarcinomas compared with normal cervical epithelia fresh tissues.

qRT-PCR data also confirmed increased levels of ezrin mRNA expression in SCC and AC compared with the normal cervical epithelia in fresh tissues (Figure 3).

### Association between ezrin overexpression and clinicopathological parameters of cervical cancers

Ezrin protein expression was significantly higher in cervical cancers with metastasis (100%, 104/104) than in cases with no metastasis (54.2%, 71/131) (*P*<0.05). Also, it was higher in poorly (94.3%, 33/35) and moderately (84.0%, 89/106) differentiated cervical cancers than in well differentiated cervical cancers (56.4%, 53/94) (*P*<0.05). For the TNM and FIGO clinical stages, the strongly positive rate of ezrin protein was 96.1% (122/127) in advanced (stages IIB–IV) cervical cancers, but only 49.1% (53/108) in early stage cases (I–IIA) (*P*<0.05). However, ezrin overexpression was not related to the histological types of cervical cancers; the strongly positive rate of ezrin protein was 76.8% (96/125) and 70.3% (71/101) in keratinizing and non-keratinizing cervical SCC, respectively (*P*>0.05) (Figure 1, Table 2).

**Table 2:** Ezrin overexpression and clinicopathological features of cervical cancers

**Clinical features**	**Cases (n)**	**Strongly positive cases *n*. (%)**	***P*-value**
**Histological types**	226		
Keratinizing	125	96 (76.8%)	NS
Non-keratinizing	101	71 (70.3%)	
**Differentiation**	235		
Poorly diff.	35	33 (94.3%)*	<0.05^***a***^
Moderately diff.	106	89 (84.0%)*	
Well diff.	94	53 (56.4%)	
**Staging**	235		
Early (I-IIA)	108	53 (49.1%)	<0.05^***b***^
IA	16	5 (31.3%)	
IB	41	22 (53.7%)	
IIA	51	26 (51.0%)	
Late (IIB-IV)	127	122 (96.1%)**	
IIB	11	9 (81.8%)	
IIIA	12	11 (91.7%)	
IIIB	46	44 (95.7%)	
IVA	41	41 (100%)	
IVB	17	17 (100%)	
**LN Metastasis**	235		
Positive	104	104 (100%)*	<0.05
Negative	131	71 (54.2%)	

Diff.: *differentiation*; NS: *not significant* *P<0.05 **P<0.01.

^***a***^*Poorly & moderately diff. vs well diff.*^***b***^*early stage (stage-IA + stage-IB + stage-IIA) vs late stage (stage-IIB + stage-III + stage-IV).*

### Evaluation of ezrin as a potential prognostic marker for early stage of cervical cancers

**Figure 4:**
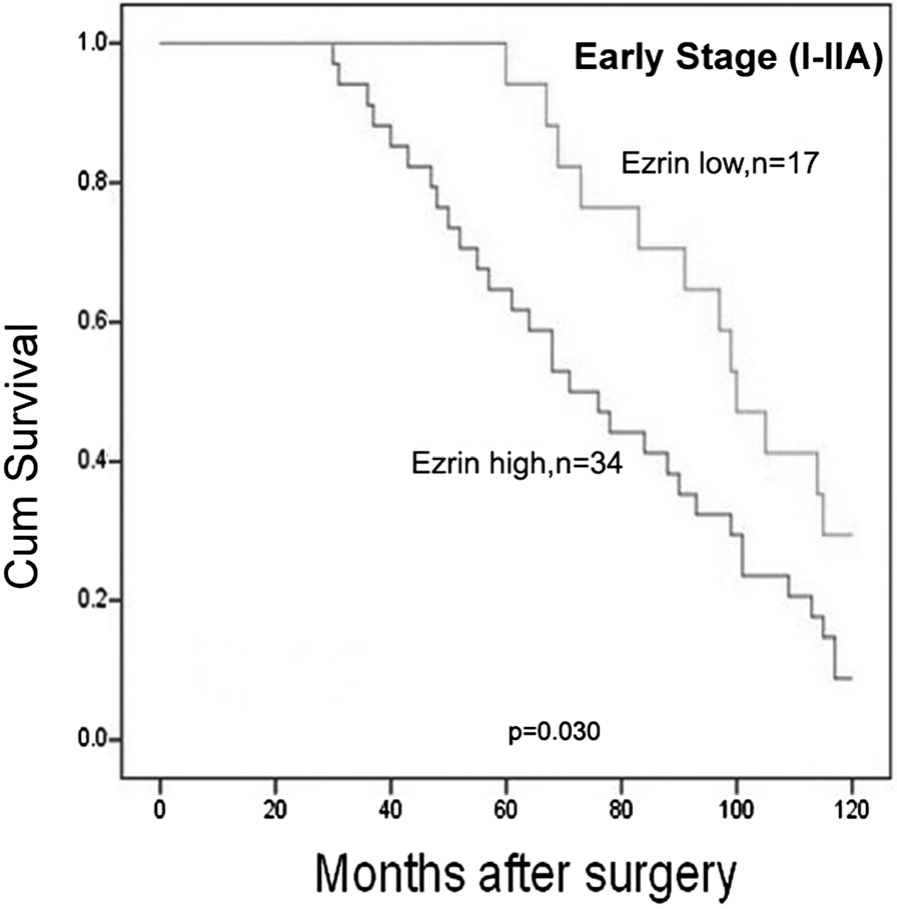
**Kaplan-Meier survival curves illustrating the significance of ezrin expression in early-stage uterine cervical cancers.** In early-stage cervical cancers (stages I–IIA, n=51), patients with high ezrin expression had significantly reduced cervical cancer-specific 10-year survival rates relative to those with low ezrin expression (log rank *P*=0.030).

A total 109 of cervical cancer patients were identified for analysis of prognostic evaluation, of which 51 were early stage cervical cancers and 58 were late stage. For early-stage (I–IIA) cervical cancer patients, the survival analysis demonstrated that high ezrin expression was associated with lower 10-year survival rate (log-rank *P*=0.030; Figure 4), but there was no significance in patients with advanced stage (IIB–IV) cervical cancers (data not shown, log-rank *P*=0.352).

### Correlation between ezrin overexpression and high-risk HPV infection in cervical cancers

All 52 cases of normal cervical epithelia were negative for high-risk HPV infection. The positive rate of high-risk HPV infection was 60.0% in CIN-1, 79.4% in CIN-2, 84.7% in CIN-3, 82.4% in CGIN, and 82.1% in cervical cancers according to HPV-DNA PCR detection. Of 235 cases of cervical cancer, 193 cases were positive for high-risk HPV infection, and 42 cases were negative. Interestingly, the strongly positive rate of ezrin protein was significantly higher in HPV-infected cervical cancers (82.9%, 160/193) than in HPV-negative cases (16.7%, 7/42) (*P*<0.01). This result may indicate a positive correlation of high-risk HPV infection and ezrin overexpression in cervical cancers. However, a statistically significant correlation between ezrin overexpression and high-risk HPV infection in the precancerous lesions was not found (Additional file 3: Table S3).

## Discussion

Despite the generally good prognosis for early stage cervical cancer patients, many affected individuals still die as a result of metastasis and recurrence, which is the major cause for most cancer-related deaths [20-23]. Therefore, the identification of reliable biomarkers for identifying cancer and predicting recurrence is critical for early diagnosis and prognostic evaluation, and for therapeutic molecular targets of cervical cancers.

The cytoskeletal organizer, ezrin, was first identified as an important metastatic regulator in rhabdomyosarcoma and osteosarcoma [13,24]. More interestingly, Arslan et al. [25] found that ezrin was concentrated at the apical (luminal) membrane surface with a weak cytoplasmic distribution in normal breast epithelial cells; however, ezrin localization was found to change distinctly with tumor stage. In early stage IA breast tumors, ezrin intracellular distribution localized with a strong perinuclear pattern and weak cytoplasmic and membrane staining. In stage IIB tumors, ezrin acquired a greater level of diffuse cytoplasmic staining and retained a weaker perinuclear and membrane distribution. In late stage IIIB large tumors, ezrin was almost exclusively confined to a diffuse cytoplasmic distribution with little evidence for membrane association or perinuclear localization in virtually all of the breast tumors. However, the distribution characteristics of ezrin in uterine cervical cancers have not yet been reported. This is the first study to our knowledge to report the significance of abnormal localization of ezrin protein in uterine cervical cancers. Here we observed that ezrin protein showed apical membranous, cytoplasmic and perinuclear staining patterns in cervical lesions. Most importantly, the apical membranous staining pattern of ezrin protein was only found in normal cervical epithelia and was associated with favorable characteristics of cervical lesions; however, the perinuclear localization of ezrin was only found in malignant epithelial tumors of the cervix, including SCC and AC. For the cytoplasmic staining pattern, ezrin was weakly positive in only 11.5% of normal cervix samples, but showed strong and diffuse staining pattern in cervical cancers (SCC and AC) and their precancerous diseases (CIN and CGIN), indicating that the altered localization of ezrin is useful in the early diagnosis of cervical cancers and precancerous diseases. Interestingly, the perinuclear localization of ezrin was observed in 51.4% of ezrin-positive cervical cancer cases, and the positive rate of perinuclear localization was higher in well (75.3%) and moderately (44.2%) differentiated cervical cancers than in poorly differentiated cases (21.2%) (*P*<0.05). Furthermore, the positive rate of perinuclear localization of ezrin was significantly higher in early stage cervical cancers compared with advanced stage cases (*P*<0.05), demonstrating that the perinuclear localization of ezrin might predict malignant potential as it was significantly associated with higher differentiation and early stage disease.

Accumulating evidences showed that ezrin protein expression was markedly increased in a variety of human cancers compared with their non-malignant tissue counterparts [17,26]. A recent study also demonstrated that high ezrin expression could promote processes involved in tumorigenesis including cell proliferation, colony formation and migration, which may preliminarily explain the reason why ezrin predicts poor prognosis in breast cancer [15,27], pancreatic cancer [11,12,28], ovarian cancers [10] and osteosarcomas [3]. Tan J et al. [29] demonstrated that ezrin mRNA and protein expression in late stage cervical cancer patients and lymph node metastasis-positive patients were significantly higher than in equivalent early stage patients through qRT-PCR and western blotting analysis of 56 cases of fresh cervical cancer tissues. In this study, we found that the positive rate of ezrin protein was higher in CIN, CGIN, SCC, and AC than normal cervical tissues; however, the strongly positive rate of ezrin protein was only higher in SCC and AC compared with the precancerous diseases and normal cervical epithelia. qRT-PCR data also confirmed increased levels of ezrin mRNA expression in SCC and AC compared with normal cervical epithelia. These findings indicate that ezrin plays an important role in tumor progression, and ezrin protein may be a useful diagnostic marker for cervical cancers and their precancerous diseases. Additionally, we found that the strongly positive rate of ezrin protein was significantly associated with the differentiation, lymph node metastatic status and clinical staging of cervical cancers. It was much higher in poorly and moderately differentiated cancers than in well differentiated cases, and it was also higher in cervical cancers with metastasis than in cases with none. Moreover, overexpression of ezrin showed a correlation with the TNM clinical stages, which is higher in advanced (stages IIB–IV) cervical cancers than in early stage cases (I–IIA). However, ezrin overexpression was not related with the histological types. Additionally, ezrin overexpression was associated with shortened 10-year survival for patients with early-stage cervical cancer (n=51, *P*=0.030), but this was not significant in patients with advanced-stage disease (data not shown, n=58, *P*=0.352), indicating that ezrin overexpression might be an effective biomarker for the prognostic evaluation of early-stage cervical cancer.

Cervical cancer is mainly caused by the presence of high-risk HPV infection [21,22]. To date, more than 200 HPV types have been reported, of which HPV16 is most common, followed generally by HPV18, HPV45, HPV31 and HPV33 [30,31]. Different countries, however, and different geographic areas in the same country have been reported to have different distribution of HPV prevalence and HPV type in cervical cancers and precancerous lesions. Guo et al. reported a very high HPV infection rate in CIN (CIN-1, 59%; CIN-2, 68%; CIN-3, 76%) and cervical carcinomas (97%) in the USA [32]. Krul et al., however, reported that the overall HPV prevalence was 82% in Surinam and 87% in the Netherlands [33]. Our previous data showed that HPV frequency was 61.7% in SCC and 60% in AC in South Korea [34]. In the present study the positive rate of high-risk HPV infection was 33.9% in CIN-1, 51.6% in CIN-2, 57.7% in CIN-3, 66.7% in CGIN, 91.7% in SCC, and 78.6% in AC of the cervix according to HPV-DNA chip. Moreover, many HPVs have been identified in healthy individuals who have no clinical symptoms, and the path from initial infection to severe epithelial lesion is still not understood in detail [35]. Recent results suggest that microRNAs play an important role in the manifestation of HPV infections in target epithelial cells [36]. In this study, we found that the strongly positive rate of ezrin protein was significantly higher in HPV-infected cervical cancers (82.9%) than in HPV-negative cases (16.7%). This result may indicate a positive correlation of HPV infection and ezrin overexpression. Auvinen et al. [37] also reported that enhanced expression of ezrin was observed in cervical HPV-associated lesions, suggesting a role in the development of cervical neoplasia and cancer. Ezrin participates in regulating cell-cell and cell extracellular matrix adhesion, thus influencing tumor cell invasion and other biological behavior. Geiger T et al. [38] found that during the very early stages of transformation in HPV16-transformed keratinocytes, many epithelial features were gradually eliminated and some mesenchymal traits emerged. Therefore, we presumed that abnormal expression of ezrin may loosen the tight junctions between cells and make the cervix more prone to HPV infection. Then, HPV infection could accelerate ezrin overexpression and lead to invasion and metastasis of cervical cancers. However, further study is needed to verify the hypothesis and to explore the mechanism by which HPV mediates the progression of the epithelial-mesenchyme transition (EMT) via ezrin in cervical tumorigenesis.

## Conclusion

In conclusion, ezrin is a potential effective predictor of poor prognosis of cervical cancer patients, especially for those with early stage disease. Determination of ezrin expression levels may help to identify high-risk cervical cancer patients and thus aid the selection of appropriate therapies.

## Competing interests

The authors declare that they have no competing interests.

## Authors’ contributions

KJ, LY, LS and JH participated in the study conception, design, case selection and experiments. LY, LS and QC carried out the data collection. QC and LZ performed the scoring of immunohistochemical staining. KJ, LYL and LZ performed the data analysis and writing of the manuscript. All the authors read and approved the final manuscript.

## Authors’ information

Zhenhua Lin is the corresponding author, and Yulin Li is the co-corresponding author.

## Pre-publication history

The pre-publication history for this paper can be accessed here:

http://www.biomedcentral.com/1471-2407/13/520/prepub

## Supplementary Material

Additional file 1: Table S1 The characteristics of ezrin protein distribution in cervical lesions. Click here for file

Additional file 2: Table S2 Correlation between ezrin perinuclear staining and clinical features of cervical cancers. Click here for file

Additional file 3: Table S3 Correlation between HPV infection and ezrin expression in cervical lesions. Click here for file
