# Supplementary material for: High expression of ezrin predicts poor prognosis in uterine cervical cancer
Source: BMC Cancer. 2013 Nov 4;13:520. doi: 10.1186/1471-2407-13-520 (PMC4228363; doi:10.1186/1471-2407-13-520)
Supplement: Additional file 1: Table S1 — The characteristics of ezrin protein distribution in cervical lesions. [file 1471-2407-13-520-S1.doc]

**Table S1** The characteristics of ezrin protein distribution in cervical lesions

| **Diagnosis** | **Total case *n*.** | **ezrin positive *n*.** | **ezrin protein staining pattern** |
| --- | --- | --- | --- |
|  |  |  | apical membranous (*n*/%) cytoplasmic (*n*/%) perinuclear (*n*/%) |
| Normal cervix | 52 | 6 | 6 (100%) 6 (100%) 0 (0) |
| CIN-1 | 65 | 53 | 0 (0) 53 (100%) 0 (0) |
| CIN-2 | 102 | 86 | 0 (0) 86 (100%) 0 (0) |
| CIN-3 | 72 | 65 | 0 (0) 65 (100%) 0 (0) |
| CGIN | 17 | 17 | 0 (0) 17 (100%) 0 (0) |
| Cancers | 235 | 210 | 0 (0) 210 (100%) 108 (51.4%) |

CIN：*Cervical Intraepithelial Neoplasia*; CGIN：*Cervical Glandular Intraepithelial Neoplasia*

Cancers: *Squamous cell carcinoma* & *Adenocarcinoma*
