# Supplementary material for: High expression of ezrin predicts poor prognosis in uterine cervical cancer
Source: BMC Cancer. 2013 Nov 4;13:520. doi: 10.1186/1471-2407-13-520 (PMC4228363; doi:10.1186/1471-2407-13-520)
Supplement: Additional file 2: Table S2 — Correlation between ezrin perinuclear staining and clinical features of cervical cancers. [file 1471-2407-13-520-S2.doc]

**Table S2** Correlation between ezrin perinuclear staining and clinical features of cervical cancers

| **Clinical features** | **ezrin positive cases (*n*)** | **Perinuclear staining (*n*/%)** | ***P* value** |
| --- | --- | --- | --- |
| **Differentiation**  Poorly diff.  Moderately diff.  Well diff. | **210**  33  104  73 | **108**  7 (21.2%)  46 (44.2%)  55 (75.3%)* | <0.05*^a^* |
| **Staging**  Early (I-IIA)  Late (IIB-IV) | **210**  87  123 | **108**  69 (79.3%)*  39 (31.7%) | <0.05*^b^* |

Diff.: *differentiation* ***a****: Poorly & moderately diff. vs Well diff.* ***b****: Early stage vs Late stage.*
