# Supplementary material for: High expression of ezrin predicts poor prognosis in uterine cervical cancer
Source: BMC Cancer. 2013 Nov 4;13:520. doi: 10.1186/1471-2407-13-520 (PMC4228363; doi:10.1186/1471-2407-13-520)
Supplement: Additional file 3: Table S3 — Correlation between HPV infection and ezrin expression in cervical lesions. [file 1471-2407-13-520-S3.doc]

**Table S3** Correlation between HPV infection and ezrin expression in cervical lesions

| **Diagnosis** | **Total *n*** | **Case *n* (%) of HPV status** | **Strongly positive rate (%)** |
| --- | --- | --- | --- |
|
| **Normal cervix**  HPV +  HPV - | 52 | 0 (0)  52 (100%) | 0 (0)  0 (0) |
| **CIN-1**  HPV +  HPV - | 65 | 39 (60.0%)  26 (40.0%) | 6 (15.4%)  3 (11.5%) |
| **CIN-2**  HPV +  HPV - | 102 | 81 (79.4%)  21 (20.6%) | 10 (12.3%)  3 (14.3%) |
| **CIN-3**  HPV +  HPV - | 72 | 61 (84.7%)  11 (15.3%) | 14 (23.0%)  2 (18.2%) |
| **CGIN**  HPV +  HPV - | 17 | 14 (82.4%)  3 (17.6%) | 5 (35.7%)  1 (33.3%) |
| **Cancers**  HPV +  HPV - | 235 | 193 (82.1%)  42 (17.9%) | 160 (82.9%)**  7 (16.7%) |

***P*<0.01

CIN：*Cervical Intraepithelial Neoplasia*; CGIN：*Cervical Glandular Intraepithelial Neoplasia*

Cancers: *Squamous cell carcinoma* & *Adenocarcinoma*
